# Progressive Motor Neuron Pathology and the Role of Astrocytes in a Human Stem Cell Model of VCP-Related ALS

**DOI:** 10.1016/j.celrep.2017.05.024

**Published:** 2017-05-30

**Authors:** Claire E. Hall, Zhi Yao, Minee Choi, Giulia E. Tyzack, Andrea Serio, Raphaelle Luisier, Jasmine Harley, Elisavet Preza, Charlie Arber, Sarah J. Crisp, P. Marc D. Watson, Dimitri M. Kullmann, Andrey Y. Abramov, Selina Wray, Russell Burley, Samantha H.Y. Loh, L. Miguel Martins, Molly M. Stevens, Nicholas M. Luscombe, Christopher R. Sibley, Andras Lakatos, Jernej Ule, Sonia Gandhi, Rickie Patani

**Affiliations:** 1 Department of Molecular Neuroscience, UCL Institute of Neurology, Queen Square, London WC1N 3BG, UK; 2 Sobell Department of Motor Neuroscience and Movement Disorders, UCL Institute of Neurology, Queen Square, London WC1N 3BG, UK; 3 Departments of Materials, Bioengineering and Biomedical Engineering at Imperial College London, Prince Consort Road, London SW7 2AZ, UK; 4 The Francis Crick Institute, 1 Midland Road, London NW1 1AT, UK; 5 UCL Genetics Institute, Department of Genetics, Evolution and Environment, University College London, Gower Street, London WC1B 6BT, UK; 6 Department of Experimental Epilepsy, UCL Institute of Neurology, Queen Square, London WC1N 3BG, UK; 7 Cerevance, 418 Cambridge Science Park, Milton Road, Cambridge CB4 0PZ, UK; 8 MRC Toxicology Unit, Lancaster Road, Leicester LE1 9HN, UK; 9 Division of Brain Sciences, Burlington Danes Building, Hammersmith Hospital Campus, Imperial College London, Du Cane Road, London W12 0NN, UK; 10 John van Geest Centre for Brain Repair, University of Cambridge, Cambridge CB2 0PY, UK

**Keywords:** amyotrophic lateral sclerosis (ALS), induced pluripotent stem cells (iPSCs), motor neurons (MNs), astrocytes (ACs), disease modeling

## Abstract

Motor neurons (MNs) and astrocytes (ACs) are implicated in the pathogenesis of amyotrophic lateral sclerosis (ALS), but their interaction and the sequence of molecular events leading to MN death remain unresolved. Here, we optimized directed differentiation of induced pluripotent stem cells (iPSCs) into highly enriched (> 85%) functional populations of spinal cord MNs and ACs. We identify significantly increased cytoplasmic TDP-43 and ER stress as primary pathogenic events in patient-specific valosin-containing protein (VCP)-mutant MNs, with secondary mitochondrial dysfunction and oxidative stress. Cumulatively, these cellular stresses result in synaptic pathology and cell death in VCP-mutant MNs. We additionally identify a cell-autonomous VCP-mutant AC survival phenotype, which is not attributable to the same molecular pathology occurring in VCP-mutant MNs. Finally, through iterative co-culture experiments, we uncover non-cell-autonomous effects of VCP-mutant ACs on both control and mutant MNs. This work elucidates molecular events and cellular interplay that could guide future therapeutic strategies in ALS.

## Highlights

• Robust and enriched motor neurogenesis and astrogliogenesis from human iPSCs
• VCP-mutant motor neurons show TDP-43 mislocalization and ER stress as early pathogenic events
• VCP-mutant astrocytes exhibit a cell-autonomous survival phenotype
• VCP-mutations perturb the ability of astrocytes to support motor neuron survival

## Graphical Abstract

**Figure:**
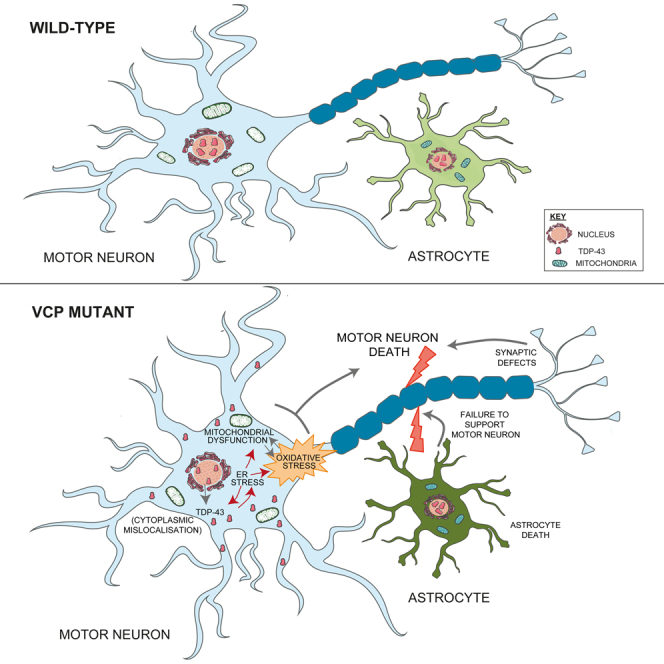

## Teaser

Hall et al. use iPSCs to examine the sequence of events by which motor neurons degenerate in a genetic form of ALS. They find that astrocytes, a type of supportive cell, also degenerate under these conditions. The ALS-causing mutation disrupts the ability of astrocytes to promote survival of motor neurons.

## Introduction

ALS (amyotrophic lateral sclerosis) is a rapidly progressive and fatal neurological condition characterized by degeneration of MNs (motor neurons). Over 20 distinct gene mutations have been identified, although their collective functions have not yet converged on a singular molecular pathway. Autosomal-dominant *VCP* mutations account for 2% of familial ALS cases (Johnson et al., 2010), comparable to mutations in the *TARDBP*-gene-encoding transactive-response DNA-binding protein, 43 kDa (TDP-43) (Sreedharan et al., 2008). The *VCP* gene encodes valosin-containing protein (VCP or p97), which is ubiquitously expressed and contributes to myriad cellular functions in a cofactor-dependent manner. VCP functions include maintenance of protein homeostasis, mitochondrial quality control, and apoptosis (Meyer et al., 2012). Wild-type TDP-43 mislocalization and aggregation form the pathological hallmark in > 95% of all ALS cases (Neumann et al., 2006), including VCP-related ALS (Johnson et al., 2010).

While undeniably valuable both animal-based models and many cell-based ones have thus far relied on VCP overexpression or knockdown in non-human or non-neuronal cells, which may fail to precisely capture the clinical pathophysiological state. Consequently, there is a need for accurate characterization of how mutant VCP affects human MNs and ACs (astrocytes). To examine early pathogenic events in VCP-related ALS, we employed patient-specific iPSCs and robust ontogeny-recapitulating methods of directed differentiation to enriched populations of both spinal cord MNs and ACs. By integrating this human experimental platform with cellular phenotyping assays, we uncovered early sequential pathogenic events in VCP-mutant MNs. Additionally, we identified VCP-mutant cell-autonomous AC pathology and a non-cell-autonomous effect of mutant ACs on both control and mutant MNs.

## Results

### Generation of iPSCs, Spinal Cord MNs, and ACs

**Figure 1:**
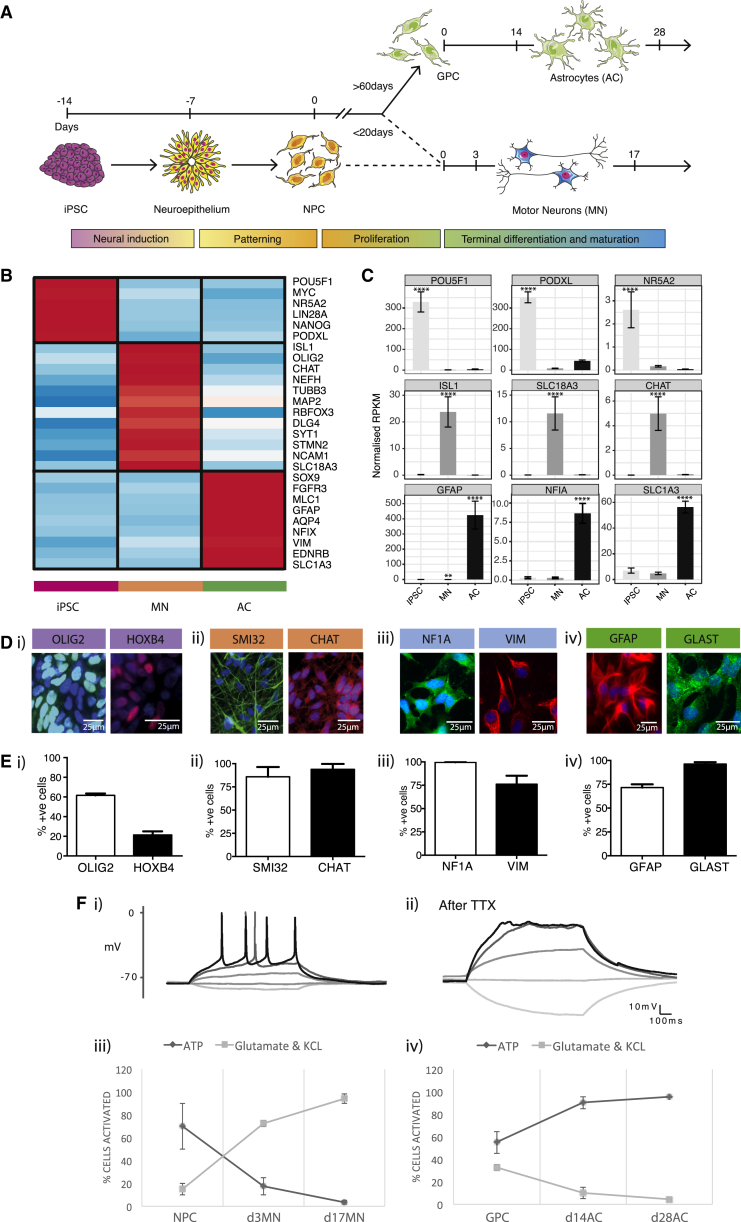
Enriched Motor Neurogenesis and Astrogliogenesis from iPSCs (A) Schematic showing differentiation strategy for motor neurogenesis and astrogliogenesis. (B) Heat map showing classic cell-type-specific gene expression from iPSC (technical n = 8, across 5 different cell lines), MN (technical n = 6, across 5 different cell lines), and AC (technical n = 8, across 4 different cell lines) cellular populations. Points represent normalized RPKM values that are log2 transformed and mean-centered. (C) Representative normalized RPKM values of cell-type specific genes (^∗∗^p < 1.0E-04 ^∗∗∗∗^p < 1.0E-08). Transcripts that showed a log 2-fold differential expression and a p value < 0.05, and that were reliably expressed in either VCP-mutant or control condition were considered as changing significantly. Error bars represent mean ± SEM. (D) Representative immunocytochemistry images of (Di) ventral spinal NPCs – Olig2 and HOXB4, (Dii) MNs – SMI32, ChAT, (Diii) GPCs – NF1A, vimentin (VIM) and (Div) ACs – GLAST, GFAP. (E) Quantitative immunocytochemistry of selected cellular markers for (Ei) ventral spinal NPCs – Olig2 and HOXB4 (Eii) MNs – SMI32, ChAT, (Eiii) GPCs – NF1A, VIM and (Eiv) ACs – GLAST, GFAP. (F) MNs generate TTX-sensitive action potentials (Fi) a representative trace recorded during current injection (−1, 0, 1, 2, 3 pA) to evoke action potentials. (Fii) Action potentials were blocked by TTX (−5, 0, 5, 10, 15 pA steps). (Fiii) 98% of our MN cultures responded to KCl and glutamate stimulation, but not to ATP. (Fiv) Enriched AC cultures were functionally validated by > 98% of the cultures demonstrating cytosolic calcium responses to ATP but not KCl. TTX = Tetrodotoxin. iPSC = induced pluripotent stem cells, NPCs = neural precursors, MN = motor neurons (d3 or d17 = after 3 or 17 days of terminal differentiation), GPCs = glial precursors, AC = astrocytes (d14 or d28 = after 14 or 28 days of terminal differentiation).

Using established reprogramming methods (Okita et al., 2011), four clones of mutant iPSCs were generated from two patients with confirmed VCP mutations: R191Q (2 clones), R155C (2 clones) (Ludtmann et al., 2017). Three healthy control iPSC lines were used as comparators (details provided in Table S1). We developed robust methods for generating highly enriched cultures of both MNs and ACs in feeder-free, chemically defined monolayer culture by adapting previously published protocols (Figure 1A) (Chen et al., 2014). RNA sequencing (Figures 1B and 1C), immunocytochemical (Figures 1D and 1E), and functional assays (Figure 1F) were used to validate our directed differentiation strategies. Neural conversion involved three small molecule inhibitors of the activin/nodal, BMP4, and GSK3β pathways for 7 days, followed by patterning with retinoic acid (RA) and a sonic hedgehog agonist (Purmorphamine) to generate spinal-cord MN precursors, which expressed OLIG2 and HOXB4 (Figures 1A, 1B, 1C, 1Di, and 1Ei). Upon terminal differentiation, these cultures yielded > 85% SMI32 and Choline acetyltransferase (ChAT) expressing MNs (Figures 1B, 1C, 1Dii, and 1Eii). Ventral spinal interneurons (INs; including V2 and V3 subtypes) only represented < 8% of our cultures by quantitative immunocytochemisty (data not shown). At day 17 of terminal differentiation (d17), whole-cell patch clamp demonstrated passive (n = 12) and active properties (n = 16) of MNs. In all cases, current injection evoked action potentials (rheobase 3.5 ± 0.5 pA) (Figure 1Fi). Spontaneous firing was also observed. 15/16 cells fired repetitively with increasing current injection. Action potentials were tetrodotoxin (TTX) sensitive (n = 3) (Figure 1Fii). We assessed the cytosolic calcium response to physiological calcium stimuli (glutamate and KCl) to confirm the presence of glutamate receptors and voltage-dependent calcium channels. 98% of d17 MNs responded to KCl and glutamate stimulation, but not to ATP (Figure 1Fiii). For AC differentiation, MN precursors were maintained in FGF2 for ≥ 60 days, yielding a population of > 80% vimentin- and > 90% NF1A-expressing gliogenic precursors (GPCs) (Figures 1A, 1B, 1C, 1Diii and 1Eiii). GPCs were terminally differentiated in BMP4 and LIF, as previously described (Gupta et al., 2012). Enriched populations of ACs expressing GLAST (glutamate aspartate transporter) (> 90%) and GFAP (glial fibrillary acidic protein) (> 70%) (Figure 1Div and 1Eiv) were functionally validated by > 98% of cells demonstrating cytosolic calcium responses to ATP but not KCl (Figure 1Fiv).

### VCP-Mutant Cultures Recapitulate Key Aspects of ALS Pathogenesis

#### Impaired Cellular Viability of VCP-Mutant MNs

**Figure 2:**
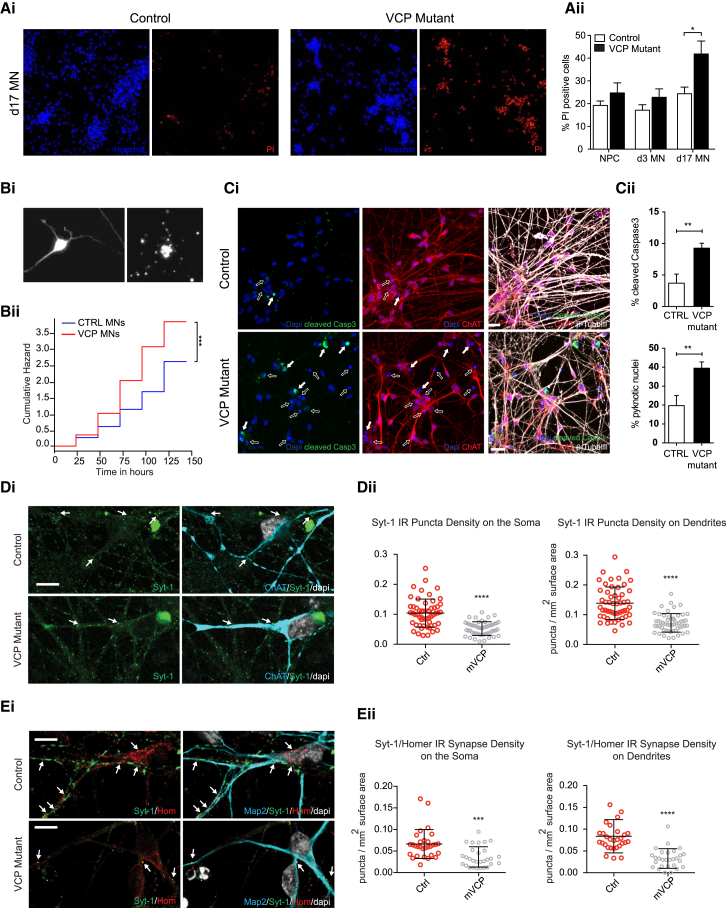
VCP-Mutant Motor Neuron Cell Survival and Synaptic Phenotypes (A) (Ai) Representative images of Hoechst (blue) and propidium iodide (PI; red) staining in control and VCP-mutant d17 MNs. (Aii) Quantification of percentage of PI-positive cells in control and VCP-mutant MNs; n = 3 control lines and 4 mutant lines for NPC, d3 MN and d17 MN; p < 0.05, unpaired t test; % cell death for each line at each time was calculated from at least two independent inductions per line on separate days and with at least three replicate wells per induction per day. At least 2,000 cells were counted for each well. Data represent mean ± SEM. (B) Longitudinal imaging based survival analysis comparing VCP-mutant versus CTRL MNs. (Bi) Representative images showing a MN captured by longitudinal imaging when alive (left panel) and dead (right panel).(Bii) VCP-mutant MNs present increased cumulative risk of death under basal conditions (cumulative hazard ratio [cHR] = 1.75, p = 1.08 × 10^−8^; CTRL MNs taken as baseline). N = 3 control lines and 3 VCP-mutant lines. To estimate survival Kaplan–Meier and cumulative risk-of-death curves were plotted using R, while Cox proportional hazards analysis, also calculated in R, was used to determine the influence of the VCP mutation on survival of MNs. The survival plot is presented with time in hours on the horizontal axis and the cumulative risk expressed as a logarithm on the vertical axis. The cumulative hazard ratio value is presented in non-logarithmic form, and is based on the control MNs, posed as 1. (C) (Ci) Representative confocal images showing apoptotic (full arrows) and pyknotic (empty arrows) cells at d17 of terminal differentiation. Scale bar: 25 μm. (Cii) Proportion of cells positive for cleaved caspase 3 (top) or with nuclear pyknosis (bottom) over total number of cells. Data are expressed as mean ± SD, ^∗∗^p < 0.01; unpaired t test. (D) Analysis of synaptic puncta in hiPSC-MNs with or without VCP mutations. (Di) Images of single planes taken by confocal microscopy, showing presynaptic synaptic marker SYT-1 (green) adjacent to the membrane of MNs (ChAT in blue) with intact nuclei (DAPI is white). (Dii) Graphs represent mean synapse density values defined by puncta per surface area on the MN soma and dendrites (N = 51-56 cells or dendrites). (E) (Ei) Images of a single confocal plane, demonstrating pre- and post-synaptic puncta labeled by SYT-1 and Homer-1 immunofluorescence, respectively. (Eii) Graphs demonstrating synaptic densities defined by proximity analysis of SYT-1 and Homer-1 positive puncta and expressed as mean number of closely adjacent puncta/cell surface area on the soma or dendrites (N = 28-30 cells or dendrites). Data represent mean ± SD. Taken together, the synaptic data in this figure were generated from 3 control lines and 3 mutant-VCP lines, each of which was technically triplicated. ^∗∗∗^p < 0.001, ^∗∗∗∗^p < 0.0001, unpaired t test. Scale Bar: 25 μm. NPC = neural precursor cells, MNs = motor neurons, d3 or d17 = after 3 or 17 days of terminal differentiation.

Percentage cell death was measured at three time points in MN development (neural precursor cells [NPCs], d3 MNs, and d17 MNs), revealing a significant increase in the VCP-mutant d17 MNs compared to control (41.8% ± 5.8% versus 20.1% ± 2.8%, p < 0.05, unpaired t test; Figure 2Aii). This was confirmed using an automated longitudinal imaging platform, cumulative hazard ratio (cHR) of VCP-mutant MNs over control: 1.75, p = 1.08 × 10^−8^, log-rank test (Figure 2B), as described previously (Barmada et al., 2010). Cell death was also assayed by measuring cleaved caspase 3 (apoptosis) and nuclear pyknosis, which were both significantly higher in VCP-mutant MNs compared to control counterparts (9.3% ± 0.8% versus 3.7% ± 1.4% cleaved caspase 3; 39.5% ± 3.3% versus 19.7% ± 5.4% pyknotic nuclei, p < 0.01, unpaired t test) (Figure 2Ci and 2Cii).

#### Synapse Formation Is Disrupted in VCP-Mutant MNs

We examined pre-synaptic puncta adjacent to soma or dendrites of d17 MNs, which were analyzed in cell clusters interconnected by axons forming a network. Pre-synaptic terminals and MNs were identified by immunolabelling for the pre-synaptic marker synaptotagmin-1 (SYT-1) and ChAT, respectively (Figure 2Di). A significant 2-fold reduction in the density of SYT-1 positive puncta on cell somata (0.052+0.003 puncta/μm2; unpaired t test, ^∗∗∗∗^p < 0.0001; Figure 2Dii left plot) and dendrites (0.073+0.004 puncta/μm2; unpaired t test, ^∗∗∗∗^p < 0.0001; Figure 2Dii right plot) was found in VCP-mutant cultures compared to control (0.104+0.006 puncta/μm2 and 0.139+0.007 puncta/μm2, respectively). To further confirm a synaptic phenotype we co-immunolabeled MNs for pre-synaptic (SYT-1) and post-synaptic (Homer-1) markers and analyzed their juxtaposition with proximity measurements. Significant reductions in synaptic density at the soma (0.036+0.004 puncta/μm2; unpaired t test, ^∗∗∗^p < 0.001) and dendrites (0.033+0.004 puncta/μm2; unpaired t test, ^∗∗∗∗^p < 0.0001; Figure 2Eii) were confirmed in VCP-mutant MNs compared to control MNs (0.066+0.006 and 0.083+0.007 puncta/μm2, respectively). To determine whether synapse loss represented early MN dysfunction or loss of interconnected MNs/Ins, we counted the number of ChAT/MAP-2 positive neurons with intact DAPI-stained nuclei in the analyzed cell clusters. Both the number of total neurons and the MN:IN ratios were comparable between the two experimental conditions, indicating that synapse loss in VCP-mutant MNs is not attributable to differential loss of MNs or INs (data not shown). Furthermore, synaptic perturbation was also revealed by transcriptional profiling of *SLITRK4, SLITRK2, CBLN2, NLGN4Y, PTPN5,* and *ACHE,* which are relevant to synapse structure and assembly (Figure S1A). Analysis of an array of different ion channels revealed further transcriptional deregulation, specifically in the delayed rectifier potassium channel, *KCNA2*; the inward rectifier potassium channel, *KCNJ5*; the sodium channel, *SCN2A;* and glutamate receptors, *GRIN2A* and *GRM7* (Figure S1B).

Having identified cell death, synapse loss, and transcriptional perturbations of genes encoding ion channels and synapse structure and/or assembly, we next addressed VCP mutation-related functional consequences by examining electrophysiological properties of our MNs on multi-electrode arrays (MEAs). Electrical activity began to appear on individual electrodes during the first few days following differentiation as random spiking. Over subsequent maturation of control MNs, uncoordinated local bursting behavior was observed on active electrodes that eventually became synchronized across the whole active network (Figure S2). These periods of high synchrony occurred at very low frequency, with an interval of 60–100 s. Within these periods of synchrony, a pattern of burst firing occurred that oscillated at a frequency of around 0.5 Hz with diminishing activity as the bursting period proceeded. VCP-mutant MNs showed an electrophysiological phenotype of an overall decrease in activity and bursting behavior, which temporally coincides with our viability and synaptic phenotypes. Furthermore, the level of functional coordination across the cell network, which resulted in the appearance of array-wide synchronized bursts, appeared to be impaired in VCP-mutant MNs (Figure S2).

#### Increased Cytoplasmic TDP-43 and ER Stress Are Early Events in VCP-Mutant MNs

**Figure 3:**
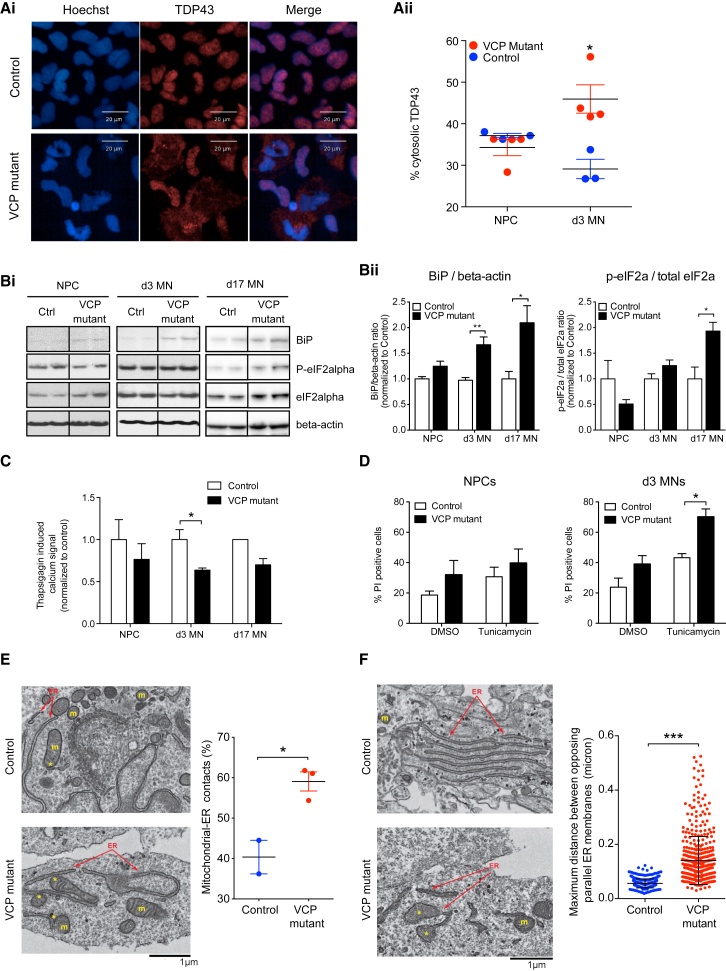
TDP-43 Mislocalization and Endoplasmic Reticulum Stress Are Early Molecular Events in VCP-Mutant Motor Neurons (A) (Ai) Immunofluorescence of control and VCP-mutant NPCs and d3 MN (blue, Hoechst; red, TDP-43). (Aii) Quantification of percentage of nuclear or cytosolic TDP-43 positive area, normalized to total TDP-43 positive area in d3 MNs (n = 3 control lines and 4 VCP-mutant lines. p < 0.05, unpaired t test; ≥ 2000 analyzed per line, each in technical triplicate). (B) (Bi and Bii) western blot images and quantification of BiP, phospho-eIF2alpha, total eIF2alpha, and β-actin levels in control and VCP-mutant NPCs, d3 MNs and d17 MNs (n = 3 control and 3 mutant clones, from at least three independent cultures per line each in technical triplicate, ^∗^p < 0.05, ^∗∗^p < 0.01, unpaired t test). (C) Histogram showing thapsigargin induced calcium signal measured in calcium free media (n = 3 control and 3 mutant clones, ^∗^p < 0.05, unpaired t test). (D) Histogram showing percentage of PI positive cells in control and VCP-mutant NPCs and d3 MNs with or without 48 hr treatment with ER stressor tunicamycin, n = 2 control lines and 3 mutant lines, p < 0.05, unpaired t test, > 50,000 cells per condition. Error bars represent mean ± SEM. (E) Representative EM images and quantification of mitochondria-ER contacts in control and VCP-mutant d17 MNs (n = 2 control lines and 3 mutant lines; n = 762 for control mitochondria and n = 1708 for VCP-mutant mitochondria; p < 0.05, unpaired t test,). Yellow asterisks show mitochondrial in contact with the ER (arrows). ER, endoplasmic reticulum; m, mitochondria. (F) Representative EM images and quantification of maximum distance between opposing parallel ER membranes (mean± SD 0.056 ± 0.0009 in control versus 0.140 ± 0.0041 in VCP-mutant, n = 375 ER from control and 463 ER from VCP mutant; p < 0.0001, unpaired t test). Yellow asterisks indicate dilated ER (arrows). ER, endoplasmic reticulum; m, mitochondria. NPC = neural precursor cells, MNs = motor neurons, d3 or d17 = after 3 or 17 days of terminal differentiation.

Mislocalization of TDP-43 from the nucleus to cytoplasm correlates with cellular toxicity (Barmada et al., 2010). To explore this known pathological hallmark of ALS in our model, we examined cytoplasmic TDP-43 during MN differentiation. NPCs revealed no significant difference between control and VCP-mutant conditions (Figure 3Ai and 3Aii). However, the percentage of cytosolic TDP-43 was significantly increased in VCP-mutant d3 MNs compared to control (46.0% ± 3.4% versus 29.1% ± 2.3% p < 0.05, unpaired t test; Figure 3Ai and3Aii). We reasoned that increased cytoplasmic TDP-43 may be linked to ER stress. We detected significantly increased expression of BiP (p < 0.01 at d3; p < 0.05 at d17) and p-eIF2alpha (p < 0.05 at d17) in the VCP-mutant MNs (Figure 3Bi and 3Bii). We examined ER calcium stores and found significant reduction in VCP-mutant d3 MNs (63.7% ± 2.4% of control, p < 0.05, unpaired t test; Figure 3C). A 48 hr tunicamycin ER stress assay also revealed significantly increased cell death in VCP-mutant d3 MNs compared to control (43.3% ± 2.7% versus 79.2% ± 5.2%, p < 0.05, unpaired t test; Figure 3D). Noting that ER stress is associated with altered contact between ER and mitochondria, we quantified mitochondrial-ER contacts using an electron microscopy (EM) approach (Schneeberger et al., 2013), which confirmed a significant increase in the VCP-mutant d17 MNs compared to control (59.1% ± 2.4% versus 40.4% ± 4.1%, p < 0.05, unpaired t test; Figure 3E). We confirmed this finding through co-immunolabeling of a mitochondrial marker (ATP5b) and an ER marker (Protein disulfide isomerase [PDI]; data not shown). Further EM analysis revealed an increase in the number of dilated ER in VCP-mutant d17 MNs compared to control (0.14 ± 0.004 versus 0.056 ± 0.00085, p < 0.0001; unpaired t test; Figure 3F). Interestingly, we found no clear evidence of transcriptional activation of the unfolded protein response, heat shock proteins, or chaperonin proteins (Figure S3A), which is consistent with the reported transient nature of these responses (Lin et al., 2007). However, we found robust transcriptional changes suggesting down-regulation of protein translation, consistent with our aforementioned evidence of VCP-mutation-dependent ER stress inducing a cytoprotective translational arrest in d3 MNs (Figures S3B and S3C). Collectively, these data suggest elevated ER stress in the VCP-mutant MNs as an early phenotype detectable in d3 MNs.

#### VCP-Mutant MNs Exhibit Decreased Mitochondrial Membrane Potential and Evidence of Oxidative Stress

Mitochondrial membrane potential (Δψ_m_) was examined using the fluorescent cationic dye TMRM (tetramethylrhodamine methyl ester) (Figure S4A). VCP-mutant MNs exhibited significantly lower Δψ_m_ at d17 (54.7% ± 6.6% versus 100%, p < 0.05, unpaired t test; Figure S4A). We examined the response of Δψ_m_ to the complex V and I inhibitors oligomycin and rotenone, respectively. Inhibition of complex I by rotenone produced a rapid loss of Δψ_m_ (Figure S4B), while inhibition of complex V had little effect, confirming intact respiration maintained Δψ_m_ in both control and VCP-mutant MNs. These findings are consistent with uncoupling of oxidative phosphorylation in VCP-mutant MNs as previously reported in other model systems (Bartolome et al., 2013). We next studied the balance of reactive oxygen species (ROS) generation and levels of endogenous antioxidant as a measure of oxidative stress. We assessed superoxide production by measuring the rate of oxidation of the dihydroethidium dye (DHE) as a ratio of the oxidized over the reduced form (Figure S4Ci). We found that the VCP-mutant d3 MNs and d17 MNs exhibited significantly higher rates of ROS production compared to control (d3 MN 189.9% ± 30.4%, d17MNs 187.3% ± 42.7%; p < 0.05, unpaired t test; Figure S4Cii). Furthermore, a significant decrease in glutathione levels was identified in the VCP-mutant d17 MNs only (50.16% ± 8.0% versus 100% control, p < 0.001, unpaired t test; Figure S4D). Together, these data suggest an early increase in ROS generation in MNs, but this is only associated with oxidative stress due to depletion of glutathione levels in d17 MNs.

#### Autonomous and Non-autonomous Effects of VCP-Mutant ACs in MN Degeneration

**Figure 4:**
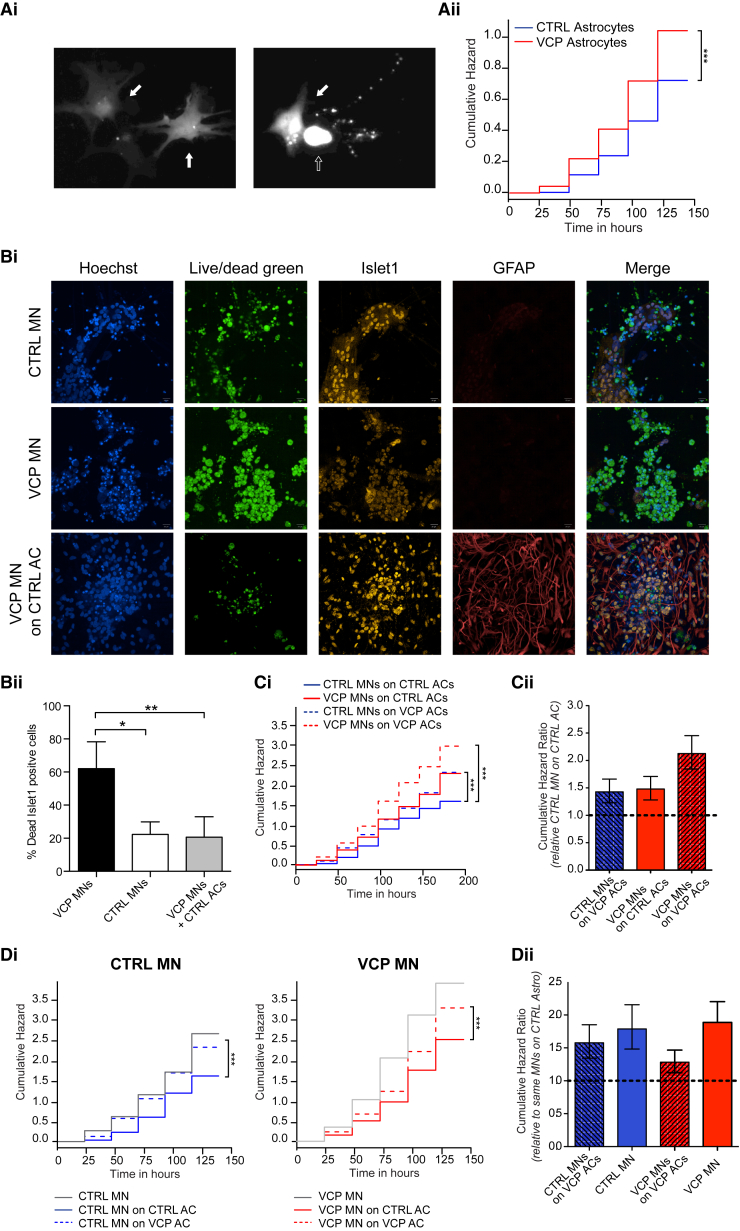
VCP-Mutant Astrocyte Cell-Autonomous and Non-Cell-Autonomous Phenotypes (A) Longitudinal-imaging-based survival analysis comparing VCP-mutant versus CTRL ACs. (Ai) Representative images showing two ACs captured by longitudinal imaging when both alive (left panel, full arrows) and after one died (empty arrow) at a later time point (right panel). (Aii) VCP-mutant ACs show increased cumulative risk of death under basal conditions (cumulative hazard ratio [cHR] = 1.5, p = 4.5 × 10^−4^; CTRL ACs taken as baseline). N = 3 control lines and 3 mutant lines. To estimate survival Kaplan–Meier and cumulative risk-of-death curves were plotted using R, while Cox proportional hazards analysis, also calculated in R, was used to determine the influence of the VCP mutation on the survival of ACs. The survival plot is presented with time in hours on the horizontal axis and the cumulative risk expressed as a logarithm on the vertical axis. The cumulative hazard ratio value is presented in non-logarithmic form, and is based on the control ACs, posed as 1. (B) MN death analysis in MN-AC co-cultures. (Bi) Representative images showing cultures of control or VCP-mutant MNs alone, or VCP-mutant MNs cultured on control ACs. Hoechst: nuclear. Live/dead green: dead cells. Islet1: MN marker. GFAP: astrocyte marker. (Bii) Quantification of % dead islet1 positive cells, p < 0.01; unpaired t test. Error bars represent mean ± SEM. (C) (Ci) Longitudinal imaging based survival analysis of different MNs and ACs co-cultures from both VCP and control lines. VCP-mutant MNs present a significantly decreased survival compared to all other culture conditions when co-cultured with VCP ACs (dashed red line). Co-cultures of control and VCP cells in any combination present a less severe, but still significantly increased, survival deficit (dashed blue line and solid red line), compared with a co-culture of control MNs and ACs (solid blue line). (Cii) Graph showing the cHR of the different co-culture paradigms, compared to survival of control MNs on control ACs set at 1 (dashed line). cHR values are: CTRL MNs on VCP-mutant ACs = 1.43, p = 3.8 × 10^−6^; VCP-mutant MNs on CTRL ACs = 1.48, p = 5.32 × 10^−7^; VCP-mutant MNs on VCP-mutant ACs = 2.13, p < 2 × 10^−16^. (D) (Di) Comparison of control (left) and VCP-mutant (right) MN survival across the different culture conditions to elucidate non-cell autonomous effect of ACs on MN survival. (Dii) Graph showing the cHR of all groups compared to survival of the same MNs when co-cultured with control ACs set at 1 (dashed line). cHR values are: CTRL MNs on VCP-mutant ACs = 1.57, p = 3.3 × 10^−8^; CTRL MNs alone = 1.77, p = 6.0 × 10^−9^; VCP-mutant MNs on CTRL ACs = 1.58, p = 3.2 × 10^−9^; VCP-mutant MNs on VCP-mutant ACs = 2.03, p < 2 × 10^−16^; VCP-mutant MNs one = 3.00, p < 2 × 10^−16^; n = 3 control lines and 3 mutant lines for MN and 2 control lines and 2 mutant lines for ACs. To estimate survival Kaplan–Meier and cumulative risk-of-death curves were plotted using R, while Cox proportional hazards analysis, also calculated in R, was used to determine the influence of VCP and co-culture conditions on survival of MNs and ACs. All survival plots are presented with time in hours at the horizontal axis and the cumulative risk expressed as a logarithm at the vertical axis. The cumulative hazard ratio values are presented in non-logarithmic form, and are all based on the relevant control for that particular experiment, posed as 1. MNs = motor neurons, ACs = astrocytes.

We next evaluated the contribution of VCP-mutant ACs in our model. Using cross-sectional end point analysis, we found no significant difference in survival between control and VCP-mutant ACs (glial precursor cells [GPCs], d14 ACs and d28ACs, p > 0.05, unpaired t test; data not shown). However, recognizing the proliferative capacity of ACs in their differentiated state, we reasoned that this might allow an underlying survival phenotype to escape detection when using a cross-sectional approach. Therefore, we again employed the more sensitive real-time longitudinal-imaging platform and indeed detected a significant increase in risk of cell death of the VCP-mutant ACs compared to control ACs (Figure 4Ai). It is noteworthy that the overall cumulative hazard ratio was lower for ACs (both control and mutant) than that seen in MN cultures.

We investigated if the same sequence of molecular events occurring in VCP-mutant MNs was also responsible for AC death. Notably, there was no increased vulnerability of GPCs, d14 ACs, or d28 ACs to an ER stressor, tunicamycin, when comparing VCP-mutant to control cultures (Figure S4E). A transient decrease in the Δψ_m_ was observed in d14 VCP-mutant ACs compared to controls (72.47% ± 2.09% versus 100%, p < 0.05, unpaired t test; Figure S4F). There was a transient significant increase in the production of ROS in the VCP-mutant GPCs versus control GPCs (p < 0.05, unpaired t test; Figure S4G), with no significant difference in glutathione levels in the control versus mutant AC lineage (Figure S4H). Taken together, our findings suggest only transient changes in mitochondrial health and ROS production, but, overall, mutant ACs at least partially differ from MNs in their underlying molecular phenotypes.

We then addressed if the VCP-mutant MN survival phenotype could be rescued by co-culture with control ACs. Using cross-sectional end-point analysis, we found that control ACs were indeed able to ameliorate the survival phenotype of VCP-mutant MNs in co-culture (76.9% ± 6.9% in VCP-mutant MNs alone, 23.6% ± 6.5% in control MNs alone, 21.9% ± 3.9% in VCP-mutant MNs co-cultured with control ACs, ^∗^p < 0.05, ^∗∗^p < 0.01; unpaired t test, Figures 4Bi and 4Bii). We next questioned if the VCP mutation perturbed this ability of ACs to promote neuronal survival in co-culture, indicating a potential non-cell-autonomous mechanism of injury in VCP-related MN death. To systematically and robustly address this, we again employed the sensitive automated longitudinal-imaging method. Using this approach, we could demonstrate that VCP-mutant ACs were less able to promote survival of both control and VCP-mutant MNs compared to control ACs (Figures 4Ci and 4Cii). These data collectively suggest VCP-mutation-related, non-cell-autonomous, AC-mediated effects through failure to support MNs. We further evaluated this by direct comparison of both VCP-mutant and control MNs in isolation and together in co-culture with either VCP-mutant or control ACs. We show that VCP-mutant MN survival is increased by co-culture with control ACs, while VCP-mutant ACs have a comparably reduced capacity to support VCP-mutant MN survival (Figures 4Di and 4Dii). Furthermore, when the same comparison is performed on control MNs, co-culture with control ACs clearly improves MN survival as expected. However, VCP-mutant ACs fail to improve survival of control MNs to the same degree as control ACs (Figures 4Di and 4Dii). These data indicate that co-culture with ACs normally supports survival of MNs, and that these support mechanisms are disrupted by the VCP mutation in ACs.

## Discussion

In this study, we were able to optimize robust directed differentiation of iPSCs into enriched spinal cord MN and AC cultures, which we comprehensively validated at transcriptional and functional levels in a stage specific manner. These efficient differentiation strategies in turn permitted a range of careful cellular and molecular phenotyping assays to systematically identify the early VCP-mutation-dependent and cell-type-specific phenotypes. While no differential vulnerability was detected at earlier stages of MN lineage restriction, we identified a robust phenotype within three days of terminal differentiation into MNs. Early cytoplasmic mislocalization of TDP-43 and ER stress are later followed by mitochondrial dysfunction, oxidative stress, reduced synaptic density, and cell death. Furthermore, by harnessing the sensitive longitudinal automated microscopy approach, we were able to additionally show a VCP-mutation-dependent survival phenotype in ACs. Using an iterative co-culture paradigm, we then provide evidence of AC-mediated non-cell-autonomous mechanisms of disease in VCP-mutation-related MN degeneration. Taken together, our findings suggest that the VCP mutation results in significant and progressive cell-autonomous MN pathology, which is exacerbated by an impaired AC-supportive capacity.

Cytoplasmic aggregates of TDP-43 are known to induce ER stress in ALS, while ER stress itself has also been proposed to drive cytoplasmic TDP-43 mislocalization (Walker et al., 2013, Sasaki, 2010). Increased mitochondrial-ER contacts occurred as secondary events in the VCP-mutant MNs, providing a possible explanation of how our primary phenotypes (TDP-43 mislocalization and ER stress) can be mechanistically linked to our secondary phenotypes: cytosolic TDP-43 generates ER stress, which next triggers increased tethering of the ER to mitochondria. In turn, this may induce mitochondrial depolarization, alterations in mitochondrial calcium and oxidative stress. Indeed, the latter is a well-recognized feature of both sporadic and familial forms of ALS (Ilieva et al., 2007, Kiskinis et al., 2014). The presence of oxidative stress in our model may further compound ER stress by leading to proteosomal failure (Sitte et al., 2000). Chronic ER stress in turn can lead to cell death by mitochondria-dependent or independent mechanisms (Lindholm et al., 2006). Our finding of a mutation-dependent reduction in synapse density could represent perturbation of pre-synaptic MN terminals, MN autosynapses, or IN interactions. These data may also reflect post-synaptic pathology in MNs. Accumulating evidence implicates early synaptic loss and a relative increase in glutaminergic synaptic activity, which could potentially lead to MN dysfunction by excitotoxicity-independent mechanisms (Ilieva et al., 2007). Previous studies have shown that increased glutamatergic activity-induced calcium influx results in ER stress and increased mitochondrial calcium uptake, depolarization, and ROS production, thus feeding into a vicious cycle (Shaw et al., 1995, Howland et al., 2002), which is consistent with our findings.

In the wider ALS landscape, the SOD1 (superoxide dismutase 1) mutation has been shown to cause a cell-autonomous survival phenotype in hiPSC-derived MNs (Kiskinis et al., 2014). The presence of a SOD1-mutation-dependent cell autonomous AC phenotype has yet to be systematically addressed. SOD1-mutant ACs, however, are known to exhibit a non-cell-autonomous phenotype, adversely affecting MNs (Di Giorgio et al., 2008). A similar effect has also been reported in sporadic ALS ACs (Haidet-Phillips et al., 2011). Conversely, a cell-autonomous astrocytopathy — in the absence of non-cell-autonomous effects — has been reported in the context of *TARDBP* mutations (Serio et al., 2013). It is noteworthy that our experiments uncover both a cell-autonomous and non-cell-autonomous role for ACs in the context of VCP mutations, therefore possibly suggesting divergent and mutation-specific glial contributions in ALS pathogenesis. Our co-culture results (specifically VCP-mutant ACs with control MNs; Figure 4Di) would indicate that non-cell-autonomous effects are largely attributable to impaired supportive capacity of VCP-mutant ACs.

In summary, our study demonstrates the potential of integrating directed differentiation of iPSCs with time-resolved phenotyping assays as a pre-clinical model to confidently identify primary molecular pathogenic events in ALS patient-derived MNs, together with cell autonomous and non-cell-autonomous contributions of mutant ACs in this context. Our findings could thus help drive the development of therapies addressing specific disease-initiating mechanisms and raise the prospect of targeting ACs as a strategy to ameliorate disease progression in ALS.

## Experimental Procedures

### Ethics Statement

Informed consent was obtained from all patients and healthy controls in this study. Experimental protocols were all carried out according to approved regulations and guidelines by UCLH’s National Hospital for Neurology and Neurosurgery and UCL’s Institute of Neurology joint research ethics committee (09/0272).

### Derivation of Human Fibroblasts and iPSC Generation

Informed consent was obtained from all patients prior to skin biopsy. Dermal fibroblasts were cultured in OptiMEM +10% FCS medium. The following episomal plasmids were transfected for iPSC generation: pCXLE hOct4 shp53, pCXLE hSK, and pCXLE hUL (Addgene), as previously reported (Okita et al., 2011). Details of the lines used in thus study are provided in Table S1. Two of the control lines used (control 2 and control 3) are commercially available and were purchased from Coriell (cat. number ND41866^∗^C) and ThermoFisher Scientific (cat. number A18945) respectively.

### Cell Culture

Induced PSCs were maintained on Geltrex (Life Technologies) with Essential 8 Medium media (Life Technologies), and passaged using EDTA (Life Technologies, 0.5mM). All cell cultures were maintained at 37°C and 5% carbon dioxide.

### Statistical Analysis

For any experiment, the average experimental unit is calculated, then the average across all the clones is taken, so the variation shown is the biological variation across biological replicates (n = 3 control clones, n = 4 mutant clones). The data are checked for normality, and we utilized an unpaired t test with post hoc correction for multiple testing.

## Author Contributions

C.E.H., Z.Y., M.C., G.E.T., S.G., and R.P. conceived and designed the experiments. C.E.H., Z.Y., M.C., G.E.T., S.G., and R.P. wrote the manuscript with contributions from all coauthors; C.E.H., R.L., N.L., and C.S. developed the computational pipelines and analyzed the RNA-seq data. S.J.C., D.M.K., and R.B. conducted and/or supervised electrophysiological profiling of MNs. A.L. performed and supervised the synaptic analyses. A.S. performed and supervised analysis of the longitudinal imaging data. S.H.Y.L. and L.M.M. conducted and analyzed electron microscopy. Project design and concepts were developed by S.G. and R.P., who supervised the work.

## Accession Numbers

The accession number for the RNA sequencing data reported in this paper is GEO: GSE98288.

## Supplemental Information

Document S1. Supplemental Experimental Procedures, Figures S1–S4, and Tables S1–S3

Document S2. Article plus Supplemental Information
