# Supplementary material for: Progressive Motor Neuron Pathology and the Role of Astrocytes in a Human Stem Cell Model of VCP-Related ALS
Source: Cell Rep. 2017 May 30;19(9):1739–49. doi: 10.1016/j.celrep.2017.05.024 (PMC5464993; doi:10.1016/j.celrep.2017.05.024)
Supplement: Document S1. Supplemental Experimental Procedures, Figures S1–S4, and Tables S1–S3 [file mmc1.pdf]

Cell Reports, Volume *19*

Supplemental Information

Progressive Motor Neuron Pathology and the Role of Astrocytes in a Human Stem Cell Model of VCP-Related ALS

Claire E. Hall, Zhi Yao, Minee Choi, Giulia E. Tyzack, Andrea Serio, Raphaelle Luisier, Jasmine Harley, Elisavet Preza, Charlie Arber, Sarah J. Crisp, P. Marc D. Watson, Dimitri M. Kullmann, Andrey Y. Abramov, Selina Wray, Russell Burley, Samantha H.Y. Loh, L. Miguel Martins, Molly M. Stevens, Nicholas M. Luscombe, Christopher R. Sibley, Andras Lakatos, Jernej Ule, Sonia Gandhi, and Rickie Patani

Supplementary Figure 1. Trancriptional evidence of VCP-mutant motor neuron perturbations in synapse structure and assembly, and ion channel expression. Related to Figure 2.

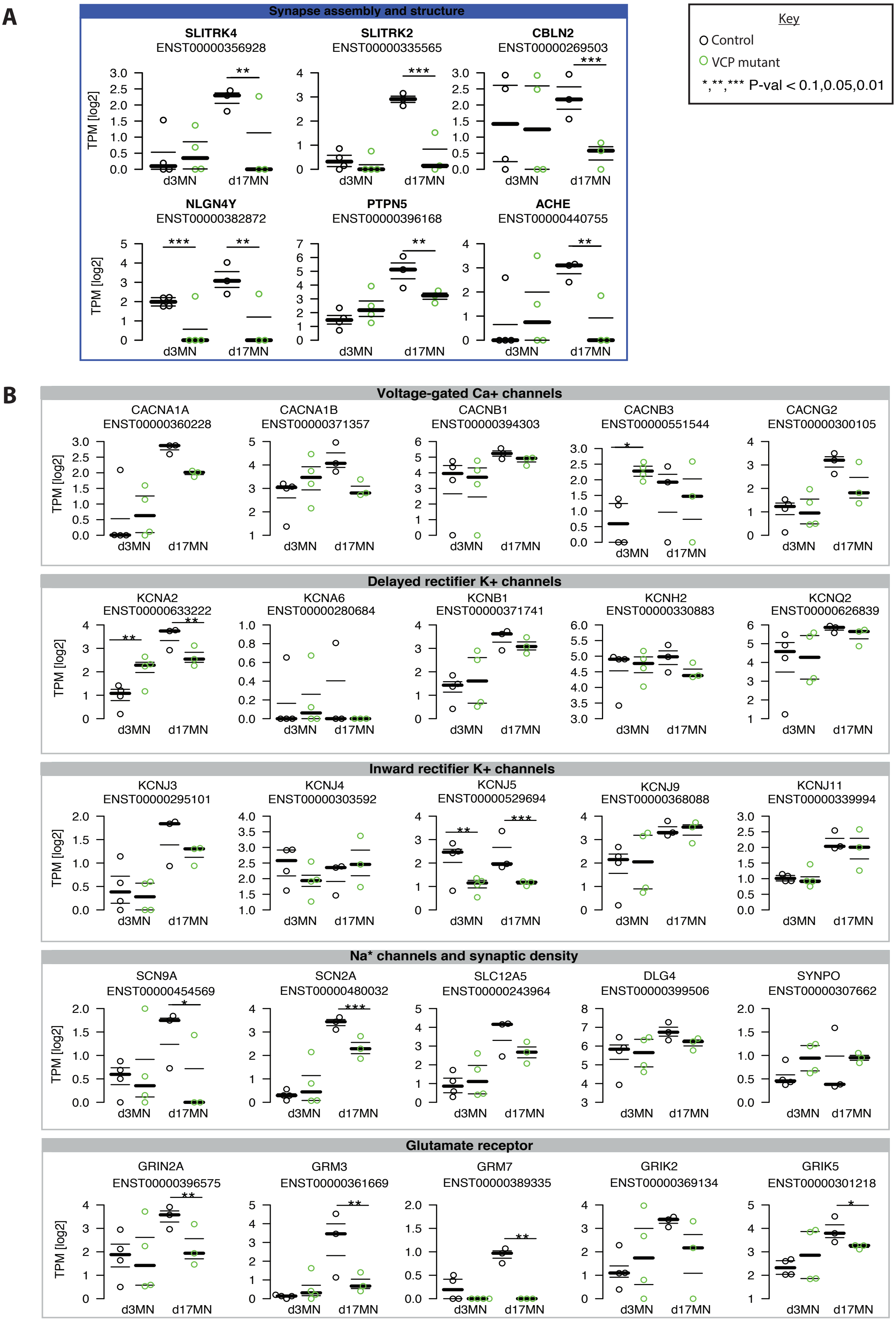

Supplementary Figure 1. (A) We provide transcriptional evidence for mutation-dependent effects on synapse assembly and structure, predominantly at a later differentiation stage (d17 motor neurons) with the following genes: SLITRK4, SLITRK2, CBLN2, NLGN4Y, PTPN5, ACHE. (B) Looking at an array of different ion channels, we find further specific evidence of perturbation, specifically in the delayed rectifier potassium channel KCNA2, the inward rectifier potassium channel KCNJ5, the sodium channel SCN2A and glutamate receptors GRIN2A and GRM7. Technical n=6, across 5 different cell lines (2 x control and 3 x VCP mutant). Transcripts that showed a log twofold differential expression and a P-value < 0.05, and that were reliably expressed in either VCP mutant or control condition were considered as changing significantly. *** = $p<0.01$,** = $p<0.05$, * = $p<0.1$ d3 MN = motor neurons after 3 days of terminal differentiation; d17 MN = motor neurons after 17 days of terminal differentiation. Grey circles = control; green circle = VCP mutant.

Supplementary Figure 2. Multi-electrode array (MEA) data suggesting a VCP-mutant motor neuron electrophysiological phenotype. Related to Figure 2.

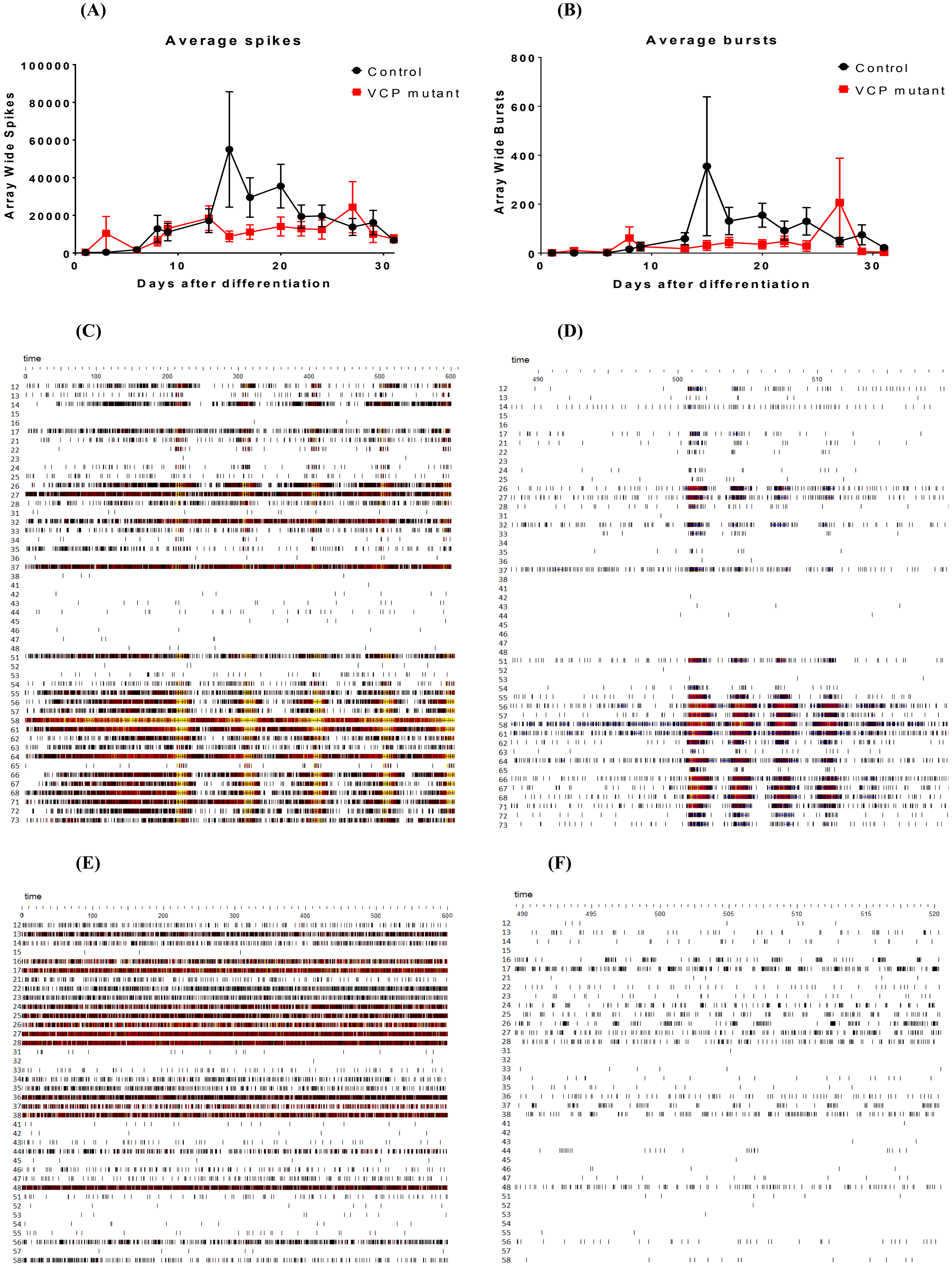

Supplementary Figure 2. MN maturation over >30 days following differentiation. (A) total spike average or (B) total burst average recorded over 600 s from a total of 23 networks derived from control or VCP-mutant cultures. (C-F) Raster plots illustrating functional activity in a typical recording in control MN network. Time is measured in seconds across the top and the electrode grid positions are shown on the vertical; (C) shows 600 seconds of recording. Recurrent periods of synchrony are clearly visible across all active electrodes. (D) shows the same data on an expanded timescale during a period of synchronous burst firing. The characteristic structure within these periods being a “hyperburst”, or burst of bursts. (E) shows equivalent data from a VCP mutant MN network. The MNs are functionally active, show little synchrony and are less ‘burst-prone’ than control MN networks. Burst firing shows less coordination (F). MN = motor neurons.

Supplementary Figure 3. Trancriptional evidence of VCP-mutant motor neuron perturbations in translation and protein complex homeostasis. Related to Figure 3.

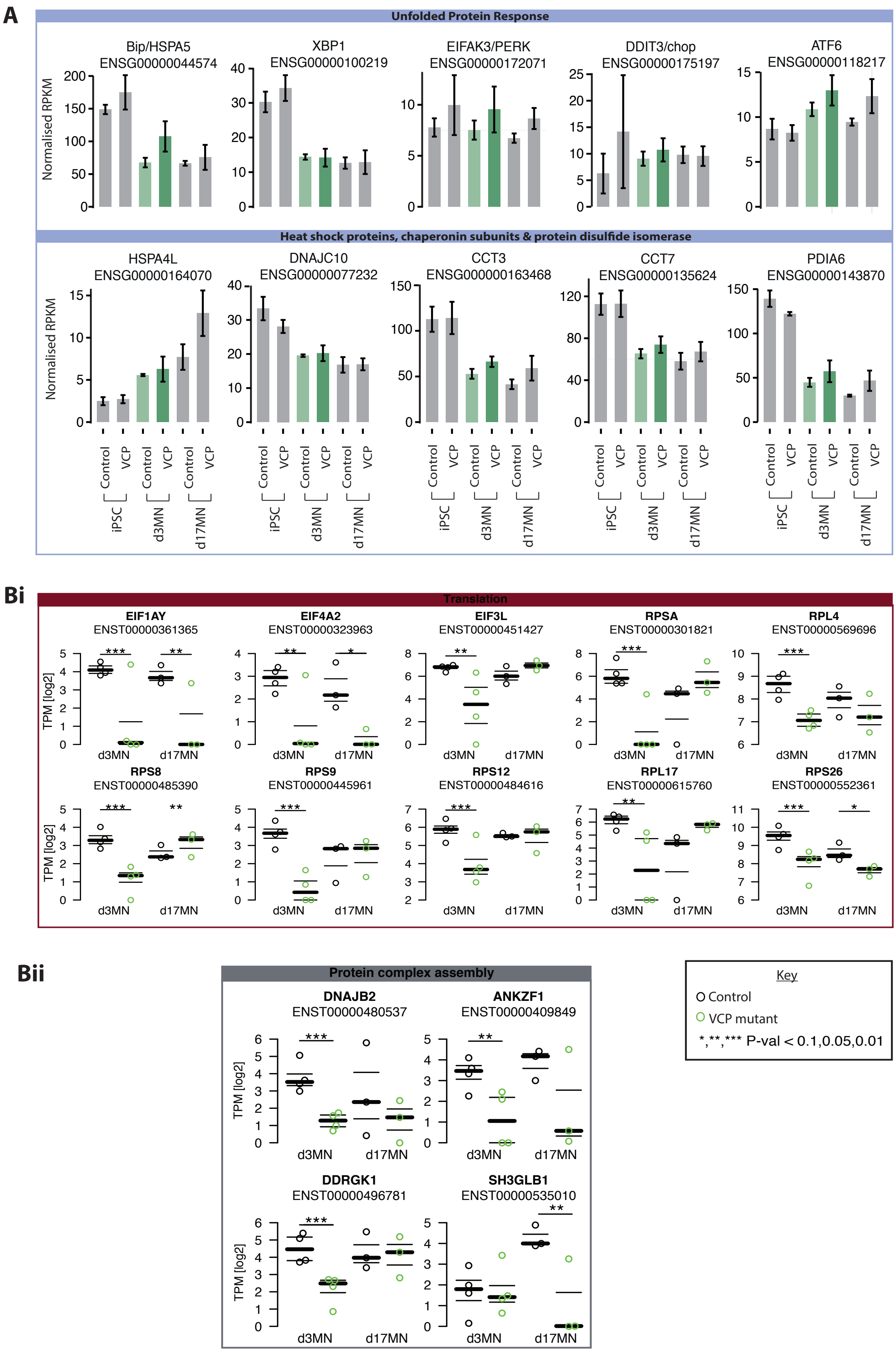

Supplementary Figure 3. (A) Representative normalized RPKM values from Control and VCP-mutant induced pluripotent stem cells (iPSC), d3 motor neurons (MNs) and d17 MNs showing selected ER stress specific genes (n=3 for each time point); UPR genes representing all 3 arms of the unfolded protein response (BIP & ATF6 = ATF6 response, XBP1 = IRE1 response, CHOP & Perk = PERK response), Heat shock proteins = HSPA4L & DNAJC10, Chaperonin subunits = CCT3 & CCT7 and Protein disulfide isomerase = PDIA6. (Bi) Differential transcript expression analysis using Kallisto and Sleuth revealed mutation-dependent changes at d3 MN stage enriched in protein translation including EIF1AY, EIF4A2, EIF3L, RPSA, RPL4, RPS8, RPS9, RPS12, RPS17 and RPS26 under 'translation' and (Bii) DNAJB2, ANKZF1, DDRGK1 and SH3GLB1 under 'protein complex homeostasis'. Technical n=6, across 5 different cell lines (2 x control and 3 x VCP mutant). Transcripts that showed a log twofold differential expression and a P-value < 0.05, and that were reliably expressed in either VCP mutant or control condition were considered as changing significantly. *** = $p<0.01$,** = $p<0.05$, * = $p<0.1$. iPSC = induced pluripotent stem cells; d3 MN = motor neurons after 3 days of terminal differentiation; d17 MN = motor neurons after 17 days of terminal differentiation. Grey circles = control; green circle = VCP mutant.

Supplementary Figure 4. VCP-mutant motor neurons exhibit decreased mitochondrial membrane potential and evidence of oxidative stress. VCP-mutant astrocytes show at least partially divergent molecular phenotypes compared to their motor neuron counterparts. Related to figures 3 and 4.

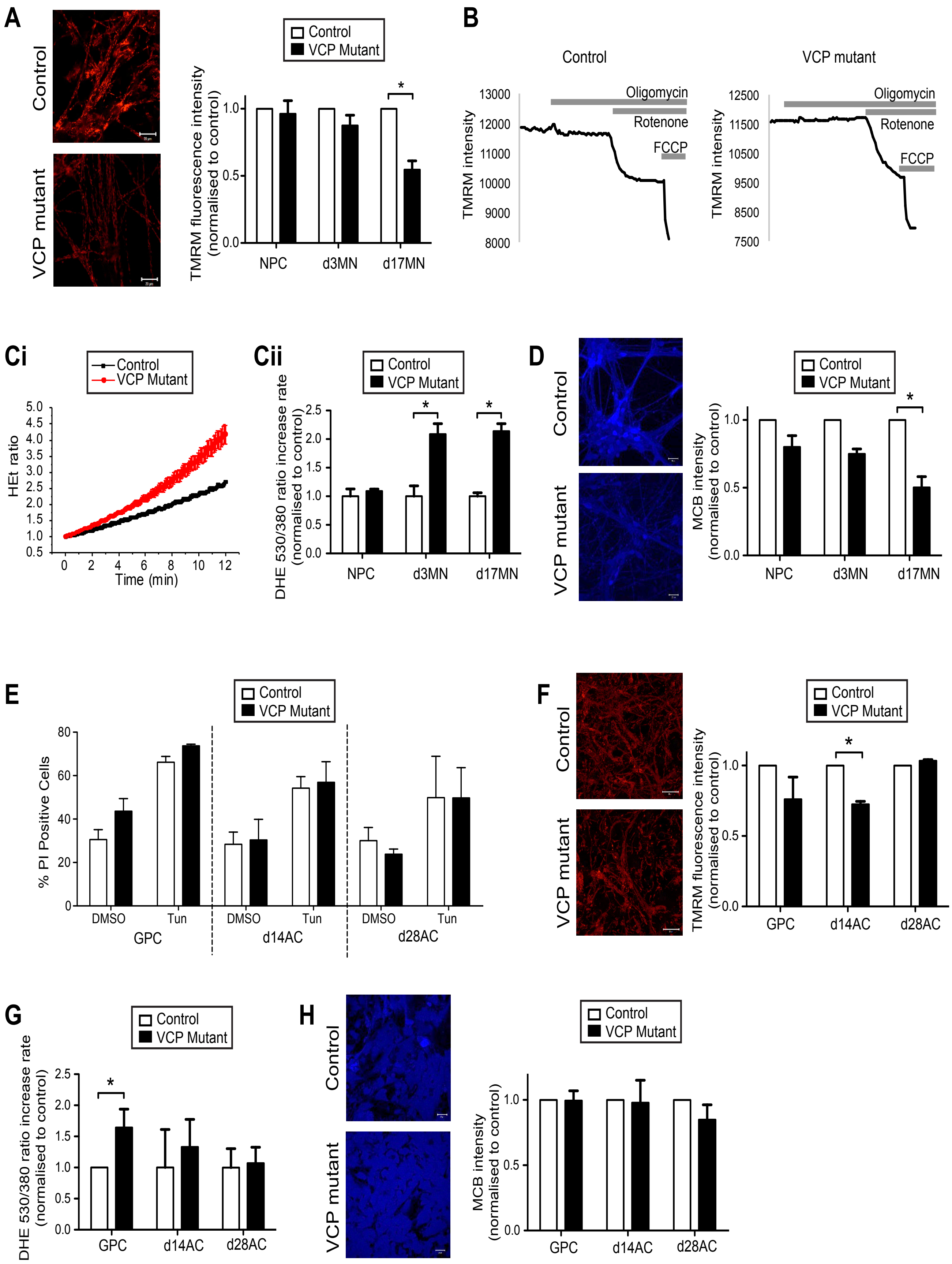

Supplementary Figure 4. A) Representative images of control and VCP-mutant d17 MNs loaded with TMRM and histogram showing basal mitochondrial membrane potential ($p < 0.05$; unpaired *t*-test). B) Representative traces showing that oligomycin does not affect mitochondrial membrane potential; rotenone induced partial depolarization; FCCP (Carbonyl cyanide-4-(trifluoromethoxy) phenylhydrazone) induced complete depolarization. Ci&ii) Representative traces and analysis showing increased ROS production rate in VCP-mutant MN lineage compared to control ($p < 0.05$, unpaired *t*-test). D) Representative images of control and VCP-mutant d17 MNs loaded with MCB, a glutathione (GSH) indicator and histogram showing decreased GSH level in VCP mutant d17 MNs but not NPCs or d3 MNs ($p < 0.05$; unpaired *t*-test). E) Tunicamycin stress assay demonstrating no vulnerability in VCP mutant ACs compared to control; unpaired *t*-test. F) Control and VCP mutant d28 ACs loaded with TMRM and histogram showing transient decreased basal mitochondrial membrane potential in VCP mutant d14 AC compared to control cells, $p<0.05$; unpaired *t*-test. G) Analysis showing ROS production rate in VCP mutant AC lineage compared to control, $p<0.05$; unpaired *t*-test. H) Representative images and histogram showing no significant differences in GSH level in VCP mutant AC lineage. N = 2 control lines and 3 mutant lines. Error bars represent mean ± SEM. NPC = neural precursor cells; d3 MN = motor neurons after 3 days of terminal differentiation; d17 MN = motor neurons after 17 days of terminal differentiation. GPC = glial precursor cells; d14 ACs = astrocytes after 14 days of terminal differentiation; d28 ACs = astrocytes after 28 days of terminal differentiation.

Supplementary Table 1.

iPSC line	Mutation present	Age of Donor	Age at disease onset	Sex of Donor
CTRL 1	None	78	N/A	Male
CTRL 2	None	64	N/A	Male
CTRL 3	None	(unknown)	N/A	Female
MUT 1	R155C	43	40	Female
MUT 2	R155C	43	40	Female
MUT 3	R191Q	42	36	Male
MUT 4	R191Q	42	36	Male

Supplementary Table 1: iPSC lines utilized in this study.

Supplementary Table 2.

**Target**	**Company**	**Catalogue number**	**Species**	**Dilution**
**Islet1**	DSHB	40.2D6	Mouse IgG1	1:50
**HoxB4**	DSHB	I12	Rat	1:50
**Olig2**	Millipore	AB9610	Rabbit	1:200
**SMI32**	Cambridge Bioscience	SMI-32R-500	Mouse IgG1	1:1000
**ChAT**	Millipore	AB144P	Goat	1:100
**3CB2 (VIM)**	DSHB	3CB2	Mouse IgM	1:5
**NFIA**	Abcam	ab41851	Rabbit	1:1000
**GFAP**	Dako	M 0761	Mouse IgG1	1:200
**GLAST**	Antibodies-online	ABIN350309	Rabbit	1:500
**TDP43**	Proteintech	10782-2-AP	Rabbit	1:500
**pTDP43, Ser409/410-1**	Cosmo Bio Ltd	CAC-TIP-PTD-P01	Rabbit	1:500
**ATP5B**	Abcam	ab14730	Mouse	1:500
**PDI**	New England Biolabs	2446	Rabbit	1:50
**Cleaved Caspase 3**	Cell Signalling Technology	9661	Rabbit	1:300
**Homer 1**	Synaptic Systems	160 003	Rabbit	1:250
**Syt1**	Synaptic Systems	clone 41.1	Mouse	1:250
**Bip**	Cell Signalling Technology	C50B12	Rabbit	1:1000
**eIF2a**	Cell Signalling Technology	#9722	Rabbit	1:1000
**Phosphor-eIF2a(ser51)**	Cell Signalling Technology	#9721	Rabbit	1:1000
**beta-actin**	Sigma	A2228	Mouse	1:8000
**MAP2**	Abcam	ab5392	Chicken	1:500

Supplementary Table 2: details of primary antibodies used in this study.

Supplementary Table 3.

	mean	s.e.m.	n
**RMP**	-57.6875	1.900346	16
**Take-off**	-45.0343	0.738535	16
**Peak**	9.062832	2.274475	16
**Amplitude**	56.51444	1.654907	16
**1/2 width**	4.1211	0.413623	16
**90-10 decay**	5.244435	0.73761	16
**AHP (mV)**	12.59701	1.30647	16
**Rheobase**	3.5	0.468718	12
**IP Resistance (Mohm)**	4784.167	726.0339	12
**Tau (ms)**	143.4867	18.79084	12
**Capacitance (pF)**	31.04523	1.661358	12

Supplementary Table 3: electrophysiological parameters are summarized here.

Supplementary methods

Motor neuron differentiation

Motor neuron (MN) differentiation was carried out using an adapted version of a previously published protocol (Chen et al., 2014). Briefly, iPSCs were first differentiated to neuroepithelium by plating to 100% confluency in chemically defined medium consisting of DMEM/F12 Glutamax, Neurobasal, L-Glutamine, N2 supplement, non essential amino acids, B27 supplement, β-mercaptoethanol (all from Life Technologies) and insulin (Sigma). Treatment with small molecules from day 0-7 was as follows: 1μM Dorsomorphin (Millipore), 2μM SB431542 (Tocris Bioscience), and 3μM CHIR99021 (Miltenyi Biotec). At day 8, the neuroepithelial layer was enzymatically dissociated using dispase (GIBCO, 1 mg/ml), plated onto laminin coated plates and next patterned for 7 days with 0.5μM retinoic acid and 1μM Purmorphamine. At day 14 spinal cord MN precursors were treated with 0.1μM Purmorphamine for a further 4 days before being terminally differentiated in 0.1 μM Compound E (Enzo Life Sciences) to promote cell cycle exit.

Astrocyte Differentiation

AC differentiation was conducted using an adapted version of a previously published protocol (Gupta et al., 2012). Briefly, iPSCs were treated identically to the motor neurogenesis protocol but underwent an additional propagation phase (>60 days) with 10ng/ml FGF-2 (Peprotech) before terminal differentiation in 10ng/ml BMP4 and 10ng/ml LIF.

Motor neuron – Astrocyte coculture

For all MN-AC co-culture experiments ACs were differentiated for a minimum of 4 weeks before being plated on 96 well plates for co-cultures. For cross-sectional endpoint analysis, NPCs were differentiated on the AC monolayer as described above for 17 days. For longitudinal imaging, MNs from the different iPSC lines were pre-differentiated for 1 week as described above, then detached using Accutase and transfected with a NEON electroporation system as described previously (Serio et al., 2013, Barmada et al., 2010). MNs were transfected with a plasmid expressing eGFP under control of cytomegalovirus (CMV) promoter, while ACs where transfected with an equivalent plasmid expressing mApple. Both plasmids were used at a concentration of 0.01 ng per cell. After transfection cells were plated in either isolation of co-culture on a laminin coated 96 well plate (TPP), and automated live imaging was performed every 24h for 7 days (single cultures) or 9 days (co-cultures) on a Zeiss Observer with incubation chamber. Culture conditions were kept as described above and medium was changed every 48h. Survival analysis was performed on the image montages assembled with Fiji to include all time points, based on the different fluorescent markers to determine viability by either cell membrane rupture or loss of fluorescence. To estimate survival Kaplan–Meier and cumulative risk-of-death curves were plotted using R, while Cox proportional hazards analysis, also calculated in R, was used to determine the influence of VCP and co-culture conditions on survival of MNs and ACs. All survival plots are presented with time in hours at the horizontal axis and the cumulative risk expressed as a logarithm at the vertical axis. The cumulative hazard ratio values are presented in non-logarithmic form, and are all based on the relevant control for that particular experiment, posed as 1.

Immunocytochemistry (ICC)

For ICC, cells were plated onto either pre-coated 8 well chamber slides (Ibidi), round glass coverslips (diameter 13mm) or clear bottom 96 well plates (Falcon). At the desired time point cells were washed once in PBS prior to being fixed in 4% paraformaldehyde for 20 minutes at room temperature (RT). Standard ICC protocols were followed. Briefly, samples were permeabilised using 0.3% Triton-X containing PBS (PBST) and blocked with either 5% normal goat serum (NGS) or 5% BSA in PBST for 60 minutes. Primary antibodies in 5% NGS / PBST were then applied overnight at 4°C. Details of primary antibodies used can be found in Supplementary Table 2. Secondary antibody incubation was performed using species specific Alexa Fluor-conjugated secondary antibodies at 1:500 dilution in 5% NGS / PBST for 60 minutes at RT. Nuclei were counterstained using either DAPI or Hoechst 33342. Cells were imaged using a Zeiss 710 confocal system with a 63x, 1.4 N.A. oil objective. Z series of images were acquired using a pinhole diameter of 1 Airy units (AU). Settings for acquisition and thresholding were kept standard for each experimental set.

RNA extraction and sequencing
The Promega Maxwell RSC simplyRNA cells kit including DNase treatment, alongside the Maxwell RSC instrument, was used for RNA extractions. The nanodrop was used to assess RNA concentration and the 260/280 ratio, and the Agilent bioanalyser was used to assess quality. RNA integrity (RIN) scores were >8 for all samples used in this work. RNAseq libraries were prepared using the Truseq stranded mRNA kit (Illumina) with 1μg input RNA. The products were then purified and enriched with PCR amplification to create the final cDNA libraries. Libraries were sent for high throughput sequencing, run for 75 cycles on a rapid flow cell, at the Institute of Neurology's NGS core facility using the Hiseq2500.

Bioinformatic analysis
RNA-seq data were generated from poly(A)+ RNA. After removing the adapter sequences and quality checking the data using FastQC, the reads were initially aligned to ribosomal RNA sequences to filter out reads that may come from ribosomal RNA contamination using bowtie2 (-v 0). The remaining reads were then aligned to the human genome (h19) using the splice aware aligner TopHat2 with default parameters. The absolute quantification of the genes was performed using HTSeqcount. The raw read count files obtained from were then processed for unsupervised clustering analysis. EdgeR package in R was used to generate normalized RPKM values. Heatmap and cell signature barplots were drawn using gplots package in R. All libraries used had <1% rRNA, <1% mtDNA, >90% strandedness and >70% exonic reads (data not shown). For differential gene and transcript expression analysis we ran Kallisto (Bray et al., 2016) and Sleuth (Pimentel et al., 2016) on a total of 8 x d3 MN (i.e. motor neurons after 3 days of terminal differentiation; 4 control and 4 VCP mutants samples) and 6 x d17 MN (i.e. motor neurons after 17 days of terminal differentiation; 3 control and 3 VCP mutants samples). Kallisto was used to 1) build a transcript index from the Ensembl GRC38 release 85 Homo sapiens transcriptome (-k 31), 2) pseudo-align the RNA-seq reads to the transcriptome and 3) quantify transcript abundances (-b 100 --single -l 275 -s 50 --rf-stranded). We next identified differentially expressed transcripts and genes with Sleuth, a companion program that uses Kallisto results to differentiate between true biological expression differences and variation resulting from sources of experimental noise. Subsequent analysis was performed with the R statistical package version 3.3.1 (2016) and Bioconductor libraries version 3.3 (R Core Team. R: A Language and Environment for Statistical Computing. Vienna, Austria: R Foundation for Statistical Computing; 2013). Prior to selecting differentially expressed genes and transcripts we identified reliably expressed transcripts and genes for each condition (VCP mutant or control immature MN or mature MN). Kallisto outputs transcript abundance, and thus we calculated the abundance of genes by summing up the estimated raw count of the constituent isoforms to obtain a single value per gene. For a given sample, the histogram of log2 gene/transcript count is generally bimodal, with the modes corresponding to non-expressed and expressed genes. Reliably expressed genes/transcripts were next identified by fitting a two-component Gaussian mixture to the log2 estimated count transcript and gene data R with package mclust (Yeung et al., 2001); a pseudocount of 1 was added before log2 transformation. A gene/transcript was considered to be reliably expressed in a given condition if the probability of it belonging to the expressed class was above 0.1 in each sample belonging to the condition. Finally genes/transcripts that showed a log twofold differential expression and a P-value < 0.05, and that were reliably expressed in either VCP mutant or control condition were considered as changing significantly.

Electrophysiology Recordings
NPCs were plated at approximately 50,000 cells/cm^2 on glass coverslips and allowed to mature for 2-3 weeks before recording. Whole-cell patch-clamp recordings were made by continuously perfusing coverslips in a recording chamber with extracellular solution containing 125 mM NaCl, 2 mM CaCl2, 1 mM MgCl2, 2.5 mM KCl, 10 mM HEPES, 30 mM glucose, pH 7.3 with NaOH, 290 mOsm, at room temperature. Neurons were visualized using an Evolve Delta EMCCD camera (Photometrics) and MultiManager software, connected to an Olympus IX73 microscope with difference interference contrast optics. Recordings were made using 5-6 MΩ pipettes filled with K-gluconate intracellular solution (126 mM K-gluconate, 4 mM NaCl, 1 mM MgSO4, 0.02 mM CaCl2, 0.1 mM BAPTA, 15 mM glucose, 5 mM HEPES, 3 mM MgATP, 0.1 mM NaGTP, pH 7.3 with KOH, 280 mOsm with sucrose), to achieve an access resistance of less than 20 MΩ. Cells showing >20% change in series resistance during the course of recording were rejected. Current clamp recordings were obtained using a MultiClamp 700B amplifier (Molecular Devices), acquired at 50 kHz and filtered at 10 kHz. Data acquisition and offline analysis were performed using LabView 2014 (National Instruments). The

calculated liquid junction potential was 15.1 mV. All values shown are corrected for liquid junction potential. After setting the bridge balance and adjusting the holding current to keep the cell at −70 mV, 1-second-long current injections, in 1-10 pA incremental steps, were delivered. A hyperpolarizing step was used to determine passive properties. Depolarizing steps were used to elicit action potentials. Parameters of action potentials were measured for the first action potential (defined as peak > 0 mV) elicited with each current injection. Values are reported as mean ± SEM. Electrophysiological parameters are summarized in Supplementary Table 3.

Multi-electrode array (MEA) analyses
Cells were plated at a density of 2×10^5 mL^{-1} on 2D arrays of 64 titanium nitride electrodes (30 µm diameter, 200 µm separation; MultiChannel Systems, Reutlingen, Germany) after coating the surface with poly-D-lysine and laminin and maintained in a water saturated environment at 37°C and 5% CO_2. Culture chambers were capped with zero evaporation lids (ALA, Scientific Instruments). Half of the media was exchanged twice weekly. Extracellular spontaneous activity was recorded at 37°C in the media environment for 10 minutes from each culture at 25 kHz and 1,100× gain (MEA1060_Inv-BC; MultiChannel Systems, Reutlingen, Germany) and high-pass filtered off-line at 200 Hz. Events were identified by threshold crossings at 5 times the standard deviation of the baseline noise using MC_Rack software (MultiChannel Systems, Reutlingen, Germany) and time stamps exported to NeuroExplorer (Nex Technologies) for further analysis. No attempt was made to discriminate multiple spikes recorded by the same electrode. Bursts were user defined as a minimum of 5 spikes separated by a maximum interspike interval of 50 ms to start the burst, a minimum duration of 50 ms and a maximum interval to end the burst of 100 ms. Synchronization was calculated by plotting the distance from each spike (timestamp) to the closest spike in a reference trace, typically the most active electrode. Recordings were carried out every 2-3 days.

Cell Survival Assays
Control and VCP mutant cells were differentiated on Geltrex-coated 96 well plate. Cultures were analyzed at 3 different developmental stages from precursors to terminally differentiated cells. Specifically, MNs were analyzed as neural precursors (NPCs) and at 3 and 17 days of terminal differentiation. Similarly ACs were analyzed as glial precursors (GPCs) and at 14 and 28 days of terminal differentiation. To determine the percentage of cell death, cells were loaded with 5ug/ml Hoechst 33342 (cell permeable nuclear dye) and 1ug/ml membrane impermeant propidium iodide (PI) for 15 minutes at room temperature in normal culture media. Image acquisition was performed using the ImageXpress system (Molecular Devices). Hoechst-stained nuclei were imaged using the 377nm excitation/530 emission filter set and images were used to determine objects for total cell counting. PI staining was imaged using the 531nm excitation/590nm emission filter set. The number of PI positive cells was determined using the multiwavelength cell-scoring module of the MetaXpress software. The threshold for counting PI positive cells was kept standard for each experimental set.

Synapse Analysis
Assessment of synaptic puncta was performed by published methods (Tyzack et al., 2014). Briefly, cultures (3-4 technical repeats) were immunolabelled for presynaptic (SYT-1), postsynaptic (Homer-1), MN (ChAT) and dendritic (MAP-2) markers. The cultures were scanned by confocal laser microscopy in 5µm depth (for SYT-1) and in 2 µm depth (for SYT-1/Homer-1). A total of 45 confocal images were collected by 1micron apart for each group. Scanning parameters were defined for the control groups and the same settings were used for the mutant iPSCs. The number of SYT-1 positive puncta adjacent to the ChAT immunoreactive soma or dendrites and also those juxtaposed to Homer-1 positive postsynaptic punctae were analyzed using an ImageJ plugin software, allowing proximity measurements (v1.0, NIH, plugin developed by B. Wark and was provided to Dr A. Lakatos by Dr C. Eroglu). Threshold values were kept the same across the optical sections. Synapse densities were defined by the number of puncta per soma or dendrite surface area.

Western blot analysis
Protein levels of a number of markers were quantified. Briefly, protein samples were extracted using to 2x laemmli buffer (4% (w/v) SDS, 20% Glycerol, 120 mM Tris-Cl (pH 6.8)). Total protein concentration was quantified using BCA assay (Pierce). Equal amount of protein samples were hen loaded and separated by SDS PAGE and transferred onto a nitrocellulose membrane. Samples were then incubated with primary antibodies overnight at 4°C followed by horseradish peroxidase conjugated secondary antibodies (Dako). The blots were imaged and the density was analyzed using Bio-Rad imaging software.

Electron microscopy

For transmission electron microscopy (TEM), d17 MNs were fixed overnight in 0.1 M sodium cacodylate buffer (pH 7.4) containing 2% paraformaldehyde and 2.5% glutaraldehyde. Samples were post-fixed for 1 h at room temperature in a solution containing 1% osmium tetroxide and 1% potassium ferrocyanide. After fixation, samples were stained en bloc with 5% aqueous uranyl acetate overnight at 4°C; the samples were then dehydrated via a series of ethanol washes and embedded in TAAB epoxy resin (TAAB Laboratories Equipment Ltd., Aldermaston, UK). Semi-thin sections were stained with toluidine blue, and areas of the sections were selected for ultramicrotomy. Ultrathin sections were stained with lead citrate and imaged using a MegaView 3 digital camera and iTEM software (Olympus Soft Imaging Solutions GmbH, Münster, Germany) in a Jeol JEM-1400 electron microscope (Jeol UK Ltd., Welwyn Garden City, UK) at an accelerating voltage of 100kV.

Measurement of mitochondrial membrane potential

Mitochondrial membrane potential was measured by loading the cells with 25nM tetramethylrhodamine methyl ester (TMRM, Life Technologies) for 40 min in physiological buffer (138 mM NaCl, 5.6 mM KCl, 4.2 mM NaHCO3, 1.2 mM NaH2PO4, 1.2 mM MgCl2, 2.6 mM CaCl2, 10 mM D-glucose and 10 mM HEPES). TMRM fluorescence signal was acquired using a Zeiss 710 confocal system with a 63x, 1.4 N.A. oil objective. Z series of images were acquired using a pinhole diameter of 1 Airy units (AU). TMRM fluorescence intensity was quantified by measuring the average TMRM fluorescence above threshold area on the maximal projection of the Z series using Metamorph software. Settings for acquisition and thresholding were kept standard for each experimental set.

Imaging of intracellular ROS production

ROS generation was measured with dihydroethidium (DHE, 10-40uM, Invirogen). All imaging was performed in physiological buffer (138 mM NaCl, 5.6 mM KCl, 4.2 mM NaHCO3, 1.2 mM NaH2PO4, 1.2 mM MgCl2, 2.6 mM CaCl2, 10 mM D-glucose and 10 mM HEPES). To avoid accumulation of oxidized products, DHE was added immediately before measuring and was present in solution throughout the experiments. Imaging was performed either with a CCD camera on an epifluorescence inverted microscope or with the FLIPR system (Molecular Devices).

Determination of GSH level

Reduced glutathione (GSH) level was measured by incubation of live cells with 40 µM monochlorobimane (MCB) for 1 hour in physiological buffer (138 mM NaCl, 5.6 mM KCl, 4.2 mM NaHCO3, 1.2 mM NaH2PO4, 1.2 mM MgCl2, 2.6 mM CaCl2, 10 mM D-glucose and 10 mM HEPES). The fluorescence signal of GS-MCB adduct was imaged using a Zeiss 710 confocal system with a 63x, 1.4 N.A. oil objective and excitation 405nm / emission at 490 nm. GS-MCB fluorescence intensity was quantified by measuring the average fluorescence intensity above threshold area on the maximal projection of the Z series using Metamorph software. Settings for acquisition and thresholding were kept standard for each experimental set.

Statistical methods for longitudinal imaging

Determination of cell-non-autonomous effects: different lines were not treated as independent groups; the dataset was analyzed as one, stratifying by biological and experimental replicate to account for behavioral variation within the single cultures, to perform the K-M survival analysis and determine the presence of a survival deficit. The effect of the genotype on survival was calculated using the Cox proportional hazard model and log-rank test, with only the genotypes considered as independent variables. This statistical approach for longitudinal imaging is based on previously published work (see references (Serio et al., 2013, Barmada et al., 2010). The graphical representation indicates the significance calculated with the Cox model, with three asterisks indicating $p<0.001$ in standard annotation (the actual p values are presented in the text).

References for supplementary information

BARMADA, S. J., SKIBINSKI, G., KORB, E., RAO, E. J., WU, J. Y. & FINKBEINER, S. 2010. Cytoplasmic mislocalization of TDP-43 is toxic to neurons and enhanced by a mutation associated with familial amyotrophic lateral sclerosis. *J Neurosci,* 30**,** 639-49.

BRAY, N. L., PIMENTEL, H., MELSTED, P. & PACHTER, L. 2016. Near-optimal probabilistic RNA-seq quantification. *Nat Biotechnol,* 34**,** 525-7.

CHEN, H., QIAN, K., DU, Z., CAO, J., PETERSEN, A., LIU, H., BLACKBOURN, L. W. T., HUANG, C. L., ERRIGO, A., YIN, Y., LU, J., AYALA, M. & ZHANG, S. C. 2014. Modeling ALS with iPSCs reveals that mutant SOD1 misregulates neurofilament balance in motor neurons. *Cell Stem Cell,* 14**,** 796-809.

GUPTA, K., PATANI, R., BAXTER, P., SERIO, A., STORY, D., TSUJITA, T., HAYES, J. D., PEDERSEN, R. A., HARDINGHAM, G. E. & CHANDRAN, S. 2012. Human embryonic stem cell derived astrocytes mediate non-cell-autonomous neuroprotection through endogenous and drug-induced mechanisms. *Cell Death Differ,* 19**,** 779-87.

PIMENTEL, H., STURMFELS, P., BRAY, N., MELSTED, P. & PACHTER, L. 2016. The Lair: a resource for exploratory analysis of published RNA-Seq data. *BMC Bioinformatics,* 17**,** 490.

SERIO, A., BILICAN, B., BARMADA, S. J., ANDO, D. M., ZHAO, C., SILLER, R., BURR, K., HAGHI, G., STORY, D., NISHIMURA, A. L., CARRASCO, M. A., PHATNANI, H. P., SHUM, C., WILMUT, I., MANIATIS, T., SHAW, C. E., FINKBEINER, S. & CHANDRAN, S. 2013. Astrocyte pathology and the absence of non-cell autonomy in an induced pluripotent stem cell model of TDP-43 proteinopathy. *Proc Natl Acad Sci U S A,* 110**,** 4697-702.

TYZACK, G. E., SITNIKOV, S., BARSON, D., ADAMS-CARR, K. L., LAU, N. K., KWOK, J. C., ZHAO, C., FRANKLIN, R. J., KARADOTTIR, R. T., FAWCETT, J. W. & LAKATOS, A. 2014. Astrocyte response to motor neuron injury promotes structural synaptic plasticity via STAT3-regulated TSP-1 expression. *Nat Commun,* 5**,** 4294.

YEUNG, K. Y., FRALEY, C., MURUA, A., RAFTERY, A. E. & RUZZO, W. L. 2001. Model-based clustering and data transformations for gene expression data. *Bioinformatics,* 17**,** 977-87.
